# The financial burden from non-communicable diseases in low- and middle-income countries: a literature review

**DOI:** 10.1186/1478-4505-11-31

**Published:** 2013-08-16

**Authors:** Hyacinthe Tchewonpi Kankeu, Priyanka Saksena, Ke Xu, David B Evans

**Affiliations:** 1 Aix-Marseille University (Aix-Marseille School of Economics), CNRS & EHESS, Centre de la Vieille Charité, 2 Rue de la Charité, 13236 Marseille, Cedex 2, France; 2 Department of Health Systems Financing, World Health Organization, Avenue Appia 20, 1211 Geneva 27, Switzerland; 3 WHO Regional Office for the Western Pacific Region, P.O. Box 2932, 1000 Manila, Philippines; 4 Department of Health Systems Financing, World Health Organization, Avenue Appia 20, 1211 Geneva 27, Switzerland

**Keywords:** Financial burden, Low- and middle-income countries, Non-communicable diseases, Review

## Abstract

Non-communicable diseases (NCDs) were previously considered to only affect high-income countries. However, they now account for a very large burden in terms of both mortality and morbidity in low- and middle-income countries (LMICs), although little is known about the impact these diseases have on households in these countries. In this paper, we present a literature review on the costs imposed by NCDs on households in LMICs. We examine both the costs of obtaining medical care and the costs associated with being unable to work, while discussing the methodological issues of particular studies. The results suggest that NCDs pose a heavy financial burden on many affected households; poor households are the most financially affected when they seek care. Medicines are usually the largest component of costs and the use of originator brand medicines leads to higher than necessary expenses. In particular, in the treatment of diabetes, insulin – when required – represents an important source of spending for patients and their families. These financial costs deter many people suffering from NCDs from seeking the care they need. The limited health insurance coverage for NCDs is reflected in the low proportions of patients claiming reimbursement and the low reimbursement rates in existing insurance schemes. The costs associated with lost income-earning opportunities are also significant for many households. Therefore, NCDs impose a substantial financial burden on many households, including the poor in low-income countries. The financial costs of obtaining care also impose insurmountable barriers to access for some people, which illustrates the urgency of improving financial risk protection in health in LMIC settings and ensuring that NCDs are taken into account in these systems. In this paper, we identify areas where further research is needed to have a better view of the costs incurred by households because of NCDs; namely, the extension of the geographical scope, the inclusion of certain diseases hitherto little studied, the introduction of a time dimension, and more comparisons with acute illnesses.

## Background

The 2010 WHO Global Status report on non-communicable diseases (NCDs) showed that they are now the most important cause of mortality worldwide. Indeed, more than 36 million people died from NCDs in 2008, mainly cardiovascular diseases (48%), cancers (21%), chronic respiratory diseases (12%), and diabetes (3%). Nearly 80% of these deaths occurred in low- and middle-income countries (LMICs), where, on average, they now exceed communicable diseases as the major cause of disease burden [1]. Even in the remaining countries where infectious diseases are the main health problem, NCDs are growing rapidly. NCDs are expected to exceed communicable, puerperal, prenatal and food diseases on the list of leading causes of death in all countries by 2020. The increasing importance of NCDs has caused them to no longer be viewed simply as a health issue but rather as a development issue worthy of discussion at a High-level Meeting of the 66^th^ General Assembly of United Nations [2].

**Figure 1:**
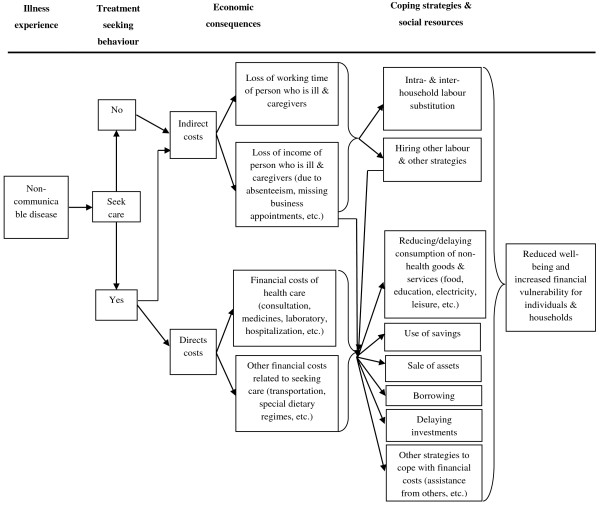
**Framework for the analysis of economic impacts of NCDs on households (modified from McIntyre et al. **[10]**).**

Considerable literature exists on the impact of NCDs on households in high-income countries [3-7]; researchers are now beginning to examine the implications of NCDs in low- and middle-income settings as well [8]. Indeed, the impact is expected to differ because there is little financial risk protection in many LMICs and thus financial costs are largely borne by households themselves rather than governments or insurance schemes [9]. The framework presented in Figure 1 describes the channels through which NCDs can affect the economic welfare of households.

We conducted a literature review to present existing evidence on the financial burden from NCDs in low- and middle-income settings, at the individual and household level. The aim is to provide accurate and relevant information on this important issue to policymakers, and determine where further research is needed.

## Methods

We performed a literature search with Cabdirect, Sciencedirect and Web of Knowledge, using combinations of the following key words: “Non-communicable disease”, “chronic illness”, “diabetes”, “cardiovascular disease”, “cancer”, and “chronic respiratory disease” with “cost”, “impoverish”, “financial burden”, “health expenditure”, “expense”, “out-of-pocket”, “health spending”, “catastrophic expenditure”, “catastrophic expense”, and “catastrophic spending”. A total of 8,966 results (including duplicates) were obtained. After duplicate removal, titles and abstracts of the remaining papers were reviewed to assess their relevance according to the following inclusion criteria: i) papers in English or French; ii) from 1990 onwards; iii) covering at least one low-, lower-middle- or upper-middle-income country^a^[11]; iv) measuring the household or individual financial costs; v) of one condition (or more) falling under the definition of “chronic diseases” [12] or classified in “Group II diseases” according to the ICD-10 code [8]. This screening led to the selection 43 articles and a secondary literature search was performed using the references cited in these selected papers. Finally, a total of 49 papers were identified, whose full-length versions were obtained for this review. Each of these studies was examined for information on disease(s), study population, analysis methods and findings. These details are presented in Additional file 1: Table S1.

## Results

### Overview of the methods used in the literature

The studies found in the literature reflect the diversity of methods used to assess household financial burden from NCDs. The methodological differences in the studies inherently prevent a formal meta-analysis from being performed. However, at the same time, these differences offer opportunities to explore results through the lens of different techniques. In this section, we present a discussion on the methodologies used.

Some studies look at a specific NCD (e.g., diabetes, cancers, cardiovascular diseases), while a majority consider NCDs in general or a combination of two or more specific NCDs. We found only one previous literature review which included studies on multiple NCDs, but it includes studies from only a few countries and did not include any studies from Africa and Latin America [13].

The original studies found also differed according to data sources and sample sizes. Some authors conducted their own surveys for the purpose of the studies, while others used data from existing surveys carried out by another entity (e.g., National Institute of Statistics, Ministry of Health, Health Insurance Plans). In these surveys, households and individuals were generally chosen randomly, through simple, stratified or cluster sampling [14-22]. However, many studies used convenient samples of patients suffering from a specific illness in health care facilities, something that we report when presenting the results [23-31]. Additionally, studies looking at specific diseases generally used relatively small samples, while those considering a broad set of diseases usually relied on bigger samples. For the assessment of diabetes costs, for example, some studies selected a small number of diabetic patients: 50 in North India, 53 in Cape Town (South Africa) and 77 in Ghana [23,25,32]. Similarly, in a study in Enugu (Nigeria), Obi and Ozumba used a sample of 95 patients suffering from cervical cancer [27]. On the other hand, up to 206,700 individuals from 48,600 households were included in a study on chronic diseases in Mexico [33]. In terms of internal validity of findings, some studies used hospital registries or insurance reimbursement records to verify the information reported by patients and/or their relatives during face-to-face interviews [34-36]; a majority of studies, however, simply accepted the answers of the respondents as being valid. Finally, some studies use data from focus group discussions and key informant interviews to complement their analyses [18,32,37-39].

In the studies looking at NCDs in general, the term “chronic diseases” is frequently used, and even if the major NCDs are usually taken into account, the definitions vary from one study to another. For example, Shi et al. defined a chronic ailment as an ailment that lasts or is expected to last for at least 12 months, resulting in functional limitations or the need for ongoing medical services, and includes disability [15]. In Kenya, Chuma et al. defined chronic illnesses as those reported to have lasted three months or more [38], while for Goudge et al., any illness that had persisted for longer than a month was defined as chronic [37]. Mondal et al. considered that a chronic illness is a condition that lasts more than three weeks, which needs to be managed on a long-term basis [40]. However, many of these studies provide the list of diseases they considered as chronic, and thus it was possible to know whether NCDs were included along with some communicable diseases (for example, HIV/AIDS). In these cases, we report results related only to chronic NCDs. Nevertheless, in some studies it was not possible to be sure that the focus was limited to only chronic NCDs.

Irrespective of the diseases considered, many studies assessing the direct costs incurred by households for the treatment of NCDs also focus on impoverishment and catastrophic health expenditure due to these expenses. Impoverishment occurs when a respondent would have had a net income above the poverty line in the absence of the expenditure on the disease, but below it after. Different poverty lines are used across studies – US$ 1 per day, US$ 1.08 per day, US$ 1.25 per day and US$ 2 per day [28,35,39,41,42].

Catastrophic heath expenditure occurs when people spend a disproportionate amount of their income (sometimes non-food expenditure) on the condition, as described in Xu et al. [43]. However, a great variety of specific definitions for catastrophic health expenditure were used in the studies presented here. The thresholds for determining a disproportionate level of expenditure vary from 10% to 60%; some studies deviated from this more standard approach. For example, Mukherjee et al. used the concept of “high health care expenditure” instead of catastrophic health payments [44]. In this study, a household was identified as having incurred high out-of-pocket expenditure on health care if its annual health care expenditure was high in comparison to those of other households within the same caste group in India [44].

### The evidence on the direct costs from non-communicable illnesses

Many of the studies assessed direct costs, which include all costs incurred by individuals and households for the treatment of NCDs. In theory, these costs should be net of any reimbursement from insurance. We present evidence on these direct costs organized by disease.

#### Diabetes

Diabetes is a leading NCD and 16 studies included in this review looked at the direct costs incurred for both outpatient and inpatient services. All studies, except one, relied on convenience samples, so the results need to be interpreted carefully. Overall, the studies found that varying shares of household income are allocated to paying for diabetes care. This ranges from as low as 5% of income for a rural low-income population in India to up to 24.5% for a low-income group in Madras (India) [34,36,45]. Spending can also differ between richer and poorer households and studies found that poorer households spend a higher proportion of their income on care for diabetes than richer households. These differences can be quite striking – one study from India found that in urban areas, the share of income spent on diabetes care in the poorest households was seven times that of the richest households [45]. Spending on diabetes can also be a considerable share of overall household health spending. A study in Sudan reported that on average 65% of household health expenditure was spent on caring for a child with diabetes [46].

Medications are frequently found to be the largest component of expenditure on diabetes [47]. Spending on medications represented from 32% to 62% of total expenditure on diabetes care in various setting such as India, Mexico, Pakistan and Sudan (Table 1). In rural Ghana, spending on insulin alone represents around 60% of the monthly income of those on the minimum daily wage [32]. Using originator-brand medication resulted in much higher spending in the only diabetes study that used random sampling rather than convenience samples. This study found that in Yemen and Mali, purchasing an originator brand medicine for glibenclamide (a medicine used to treat type II diabetes) in the private sector was found to potentially impoverish an additional 22% and 29% of the population, respectively, versus 3% and 19%, respectively, if the lowest priced generic product was purchased [41]. Laboratory and transportation costs were generally the second largest component of expenditure. Some studies also document expenditure related to special dietary regimes (up to 20% of the direct costs in North India [23]).

**Table 1:** Shares of diabetes expenditure spent on medications

Authors^*^	**Countries**	**Spending on medicines as a percentage of total expenditure on diabetes (%)**
Rayappa et al. [48]	India	32
Elrayah et al. [46]	Sudan	36 (only insulin)
Villarreal-Ríos et al. [49]	Mexico	37
Khowaja et al. [50]	Pakistan	46
Grover et al. [23]	India	62

^*^All these papers are based on convenience samples.

The presence of complications and the duration of the illness are usually associated with an increase of the direct costs. For example, Khowaja et al. found that in Pakistan, the direct cost for patients with co-morbidities was 45% higher than the direct cost for patients without co-morbidities [50]. Similarly, in India, those without complications were found to have an 18% lower cost compared to the mean annual cost for outpatient care for all patients with diabetes, while those with three or more complications had a 48% higher cost [51]. Similar results were found in India, China, Thailand and Malaysia [34,36,45,48]. These studies also highlight the fact that treatment at an early stage is much cheaper for households than treatment at a later stage with complications.

Some studies looked at coping strategies used by households to pay for these direct costs. In India, the majority of patients (89%) used their household income to fund the monitoring and treatment of their diabetes, while household savings were used by 22% of retired patients and by 19% of those in the lowest income bracket. When faced with hospitalization, 56% of patients had to dip into their savings or borrow in order to fund the costs [51]. Additionally, very few households are reimbursed by insurance. In India, Kapur found that only 1% of patients claimed the costs of treatment on insurance [51], while Ramachandran et al. observed that medical reimbursement was obtained by 14.2% of urban patients but by only 3.2% of rural patients [45]. Moreover, Khowaja et al. found that in Pakistan, none of the persons with diabetes indicated that their cost was borne by an insurance company or their employer [50].

#### *Cardiovascular diseases*

Five studies examined spending on cardiovascular diseases. In a study using data from a household survey in Kazakhstan, people with cardiac problems were found to pay on average 24% more for health care than people with other health problems [22]. As with diabetes, studies from Congo and Uganda also found that the use of originator brand drugs increases spending on cardiovascular diseases [24,41]. Once again, there was only one cardiovascular disease study that did not use a convenience sample [41].

Out-of-pocket payments for the treatment of cardiovascular diseases also lead to significant costs for households. Up to 71% of patients who had experienced an acute stroke were found to face catastrophic health expenditure^b^ in China, while 37% of them fell below the poverty line of US$ 1 per day after paying for their health care [35]. The study of Heeley et al. also found that catastrophic payments and impoverishment due to cardiovascular diseases are more common in people with no health insurance than in those with health insurance [35].

In a study covering 35 states and union territories in India, Rao et al. investigated the coping strategies used by households to deal with expenses incurred for hospitalizations due to cardiovascular diseases [52]; 57% of these expenses were paid from household savings, 35% from borrowings, and 8% from the sale of assets. In the poorest group, up to 55% of out-of-pocket spending was financed through borrowings, and only 38% through savings [52].

#### *Cancer*

Cancers also represent an emerging health problem in LMICs and seeking health care for these diseases can have a significant effect on families’ welfare. We found three papers which focus specifically on the direct cost from cancers. In a study using data from a randomized household survey in Pakistan, 27.1% of those who sought care for cancer at private facilities were found to finance their care through unsecured loans, while 7.1% relied on assistance from others [53].

Two studies using convenience samples also shed some light on components of spending on cancer care. Indeed, Zhou et al. found that health insurance facilitates the financial access of treatment for patients suffering from oesophageal cancer in China, particularly for purchasing drugs [31]. Meanwhile, transportation, multiple investigations, radiotherapy and chemotherapy were the main components of direct costs for cervical cancer in Nigeria [27].

#### *Other non-communicable diseases*

The financial burden from other NCDs, such as epilepsy, cirrhosis, chronic obstructive pulmonary disease (COPD), rhinitis and depressive disorders, is also estimated in some studies. Even if they are not as studied as the major NCDs presented previously, these types of illnesses can also exert a considerable pressure on household finances. For example, a study from Mumbai (India) based on a random sample of households found that the share of the annual personal income spent on outpatient care for allergic rhinitis was 1.7% when treatment was sought in public facilities. Similarly, care for COPD represented 13.3% of annual personal income among those using private facilities. With hospitalization at public facilities, out-of-pocket payments for COPD represented up to 62.3% of the annual personal income compared to 50.7% for hospitalization in private facilities [54]. Using a focus group, Russell and Gilson document the case of an individual suffering from asthma, who incurred a direct cost representing 15% of his monthly wage when seeking care for a sore chest in a private clinic and pharmacy [39]. Multiple laboratory tests and the presence of complications were also found to cause high expenses for a convenience sample of patients suffering from cirrhosis in Brazzaville (Congo) [26].

Coping strategies used to pay for care associated with these NCDs are similar to those used to cope with more documented NCDs. In Pakistan for example, Mahmood and Ali Mubashir using a random sample found that 22.9% of patients with circulatory diseases (heart diseases, rheumatic fever and blood pressure) who visited private doctors/clinics for treatment financed care through unsecured loans, while 8.8% relied on assistance from others [53]. Among those who did not visit any facility, 67.4% reported financial constraints as the reason for not seeking care.

#### *Non-communicable diseases combined*

We found a large number of studies – all based on randomized household surveys – looking at NCDs in general, instead of focusing on specific illnesses. Some studies highlight the association of having a household member suffering from a chronic disease with a significant increase in health care expenditure and a higher risk of impoverishment. In Russia, for example, each additional case of chronic disease in a household was found to increase the probability of incurring health care expenditure by 8% and the amount of healthcare expenditure by 6.2% [19]. Similarly, in Uganda, households with a member suffering from a chronic illness were found to be three times more likely to incur costs for health care than other households [18]. In Kazakhstan, people with chronic illness were found to pay on average 18% more than people with other health problems, while in Georgia, the mean cost for outpatient care in case of chronic illness was almost two times higher than in case of acute illness [21,22]. On the other hand, a study from India found that the relative importance of chronic diseases for spending may be lower – the mean annual per capita health expenditure for a chronic episode was 11% lower than for an acute one [44].

Undeniably, expenses incurred when seeking health care for chronic diseases represent an important financial burden for households as presented in Table 2. In fact, the costs of health care for chronic illnesses were found to represent from 5.0% of household income in rural Kenya to up to 30–50% of monthly income for vulnerable households in South Africa, where care for these illnesses were unaffordable without gifts from social networks [37,38]. Similarly, household spending on chronic illness represented 4.14% of household’s total annual health care expenditure in urban areas and 5.73% in rural areas of West Bengal in India; however, it was up to 11% in Vietnam and 32% in Maharashtra, Bihar and Tamil Nadu states of India, with a higher share for hospitalization and drugs [20,40,55]. All these studies used a random sample. Another proxy of households’ capacity to pay used in the literature is their non-food expenditure. Sun et al. found that in China, the average proportion of chronic disease expenditure to annual non-food expenditure was about 27% in Shandong Province and 35% in Ningxia province for patients covered by New Cooperative Medical Scheme (NCMS), a public health insurance scheme for rural residents [16]. For non-NCMS members, these proportions were 47% and 42%, respectively.

**Table 2:** Expenditure on chronic diseases

**Authors**^**#**^	**Countries**	**Spending on chronic illnesses as a percentage of household income (%)**	**Spending on chronic illnesses as a percentage of household total health expenditure (%)**	**Spending on chronic illnesses as a percentage of household non-food expenditure (%)**
Chuma et al. [38]	Kenya (rural)	Urban: 5.7		
		Rural: 5		
Goudge et al. [37]	South Africa (Vulnerable households)	30–50		
Mondal et al. [40]	India (West Bengal)		Urban: 4.14	
			Rural: 5.73	
Thuan et al. [20]	Vietnam		27.7^*^ (curative)	
			11.1^**^	
			58.6^***^	
Dror et al. [55]	India (Maharashtra, Bihar and Tamil Nadu states )		32	
Sun et al. [16]	China (Shandong province)			NCMS: 27
				Non-NCMS: 47
Sun et al. [16]	China (Ningxia province)			NCMS: 35
				Non-NCMS: 42

^#^All these studies used randomized samples. ^*^For all households. ^**^For households which had catastrophic health care expenditure. ^***^For households having health expenditure between 30% and 40% of their capacity to pay.

In several studies, the presence of household members with chronic ailments was also found to lead to catastrophic health expenditure and impoverishment. The probability of catastrophic expenditure was then 4.4 times higher among households having incurred expenses for treating chronically ill persons in Georgia, and up to 7.8 times higher in Burkina-Faso [17,56]. Similar results were found in West Bengal (India), in Lebanon and in China [15,40,57,58]. Up to 11.6% of households in Western and Central China were pushed under the US$ 1.08 poverty line after incurring outpatient expenses associated with chronic diseases [42]. Moreover, Shi et al. found the incidence of medical impoverishment to reach 19.6% in households where more than 50% of members had a chronic illness [16].

As with diabetes, when households are covered by health insurance, the reimbursement rates for chronic diseases are relatively low. In Shandong and Ningxia in China, for example, only 11.16% and 8.67%, respectively, of overall medical expenditure for chronic diseases was reimbursed by the NCMS [16]. However, another study from Western China found that health insurance provided protection against impoverishment due to expenses for chronic diseases [42]. Government subsidies for medicines were also found to lower the expenses for many chronic diseases in Vietnam [29].

Coping strategies documented in the literature combining chronic diseases are similar to those described in the studies on specific NCDs. In Georgia, when households were lacking financial means, the most dominant strategy was to borrow from a friend or relative (70%), followed by selling household valuables (10%) and/or household goods/products (10%) [21].

### Literature on the indirect costs due to non-communicable diseases in low- and middle-income countries

Households and individuals also bear indirect costs when they are affected by NCDs. These costs mainly include time and productivity loss by patients and caregivers because of the illness as well as income lost by patients and family members. Whereas there is no doubt that these indirect costs can pose a substantial burden on households, there are numerous methodological challenges in measuring this burden adequately; these challenges have been discussed in detail in a previous study [59]. Nonetheless, in this section, we present the available evidence on the indirect costs of NCDs as reported in the literature. This constitutes findings from 11 studies, which mainly use convenience samples, on loss of income, loss of time and other forms of financial loss related to these illnesses. We discuss possible limitations of these findings in the discussion section.

#### *Loss of income*

In India, one study suggests that the indirect cost for diabetes patients and their caregivers was 28.76% of the total treatment cost. It was claimed that loss of income of the patient comprised the greatest portion of indirect costs (60.54%), followed by loss of income of caregivers (39.46%) [23]. Rayappa et al. found that in Bangalore (India), 30.9% of respondents suffering from diabetes reported a change in personal income, and on average, they faced a reduction of 20.9% of their personal income [48]. In addition, 20.8% of the respondents reported a change in family income, with a mean reduction of 17.4%. Similarly, Arrossi et al. found that in Argentina, 39% of households with a member suffering from cervical cancer lost family income, partially or totally [28]. Among households that lost income, 47% lost less than 25% of family income, 34% lost 25–50% and 19% lost 50% or more of their income. As a result of the reported loss of income, it was estimated that the proportion of patient’s households living in poverty increased from 45% to 53%. Likewise, Obi and Ozumba found that in Nigeria, all patients suffering from cervical cancer and their relatives lost income from workplaces due to absenteeism, disengagement from work and missing business appointments [27]. In a study covering 19 countries, one of the two studies using randomized household survey data documenting indirect costs, Levinson et al. found that serious mental illness was associated with a potential reduction in earnings of 10.9% of average national earnings in LMICs [60]. The second study using randomized household survey data was from Russia and found that labour income decreased by 4.8% per additional case of chronic disease in the household [19]. Some studies only estimate the NCDs-related indirect costs for patients and their families in absolute value (local currencies or US$) [50,51,61].

#### *Loss of working time*

The loss of income borne by patients suffering from NCDs is mainly due to self-reported absenteeism from usual economic activity. In fact, the treatment of NCDs usually requires repetitive visits to health facilities in addition to the inability to work due to their poor health. This can lead to additional losses of working time both for patients and caregivers. In the literature, the mean loss of working time reported by patients was found to vary from 2.8 ± 1.7 hours per visit for diabetes in Pakistan to 58 ± 105 days per year for epilepsy in India [30,50]. Episodes of respiratory diseases can also cause important losses of working time as shown in a case study in Colombo (Sri Lanka) where Russell and Gilson found a patient suffering from asthma took two days off work for a sore chest, losing 6% of his monthly wage [39].

However, time costs are not limited to patients, but also affect caregivers. In Buenos Aires (Argentina) for example, Arrossi et al. found that in 45% of households with a member suffering from cervical cancer, at least one member reduced his/her working hours [28]. For diabetes patients in Thailand, caregivers were found to spend on average 42.21 ±39.94 hours per month on health care activities – e.g., giving medicines – and 21.87 ± 31.81 hours on activities of daily living – e.g., helping with eating and dressing [61].

#### *Other forms of indirect costs*

Some other forms of indirect costs due to NCDs were found in the literature; these generally concern households’ livelihood and welfare. The study on cervical cancer in Buenos Aires (Argentina) by Arrossi et al. examined these and also found that due to a loss of income, there were delays in payments for essential services such as telephone or electricity and as a result 43% of households had the service cut [28].

There were also significant effects on self-reported daily food consumption, which was reduced in 37% of households, while 38% of households reported that they sold property or used savings to offset income loss. Some impacts on education were found and school absences were more prevalent in 28% of households. There were also problems to pay for education in 23% of households. Furthermore, 45% of patients were cared for by one or more informal caregivers that did not live with them and one-third of these caregivers’ households reduced their daily consumption of food and 26% had delays in payments of essential services such as electricity or telephone services. It should be noted that these are the types of welfare losses have shaped the concept of catastrophic health expenditure.

There were also direct impacts on employment and at least one member stopped working in 28% of households affected by cervical cancer. Several interviewees who stopped working expressed the hope of going back to their jobs after treatment, fearing at the same time that this would no longer be possible. Similarly, a study from Bangalore (India) by Rayappa et al. found that only 33.4% of diabetes patients worked and among those working, 23% experienced problems at their job, affecting their productivity and at times requiring changing work to a less strenuous job (5.9%) or giving up the job (14.7%) [48]. Considering NCDs in general, Abegunde and Stanciole found that in Russia, chronic illnesses, which included NCDs, impose a reduction of 5% in household consumption of non-health-related items [19].

## Discussion

This literature review has presented the available evidence on the household financial burden related to NCDs in LMICs. However, before discussing its most important results, it is important to highlight some of the methodological issues in many of the studies that were included. First, the heavy reliance on convenience samples taken from people who are seeking and obtaining treatment, often at hospitals, will almost certainly result in an upward bias in costs for the average person with the condition. The people who do not seek treatment or who seek treatment at a lower level of care, implying lower costs, have no chance of being selected.

Second, self-reported costs, even from random samples of patients, are likely to be biased upwards when there are no controls. Some of the people with the condition would have incurred some health expenses in any case and this can only be captured by including controls without the condition [59,62]. In other words, it is likely that part of the costs reported by patients with NCDs were not directly associated with those conditions.

This issue is particularly important when considering indirect costs. It is clear that the method of asking people how many days they could not work overestimates the true loss in work time from a disease because many of the people, particularly in low-income countries, would not have been working on those days, or for all of those days, in the absence of the disease [59]. Nor do the studies consider whether absent workers are replaced by other family members in family enterprises or farms. For example, frequently other family members fill in for a sick person during the planting season in agriculture so that the same area of land is planted despite the illness [63]. This does not, of course, mean that there are no opportunity costs associated with the illness, but that the measured production from the family enterprise is not altered as much. In general, therefore, we expect that the costs from studies with no controls to be overestimates of both direct and indirect costs.

The substantial variations in study designs and definitions described earlier also make comparisons tricky and meta-analysis infeasible. There is considerable heterogeneity in objectives and the methodologies used in the papers. While we have more confidence in the studies relying on randomized samples, we present more details about each study in file 1: Table S1 to give readers further information and to allow them to consider possible generalizations of the results. Taking into consideration the methodological issues highlighted here and in earlier sections, we can still conclude that NCDs already impose substantial financial costs on some of their sufferers in lower-income countries. As a result, the cost of obtaining treatment for NCDs is also becoming a cause of impoverishment and financial catastrophe in these countries. While this is not particularly surprising given the growing burden of disease associated with these conditions, it has not been documented before.

Again not surprisingly, complications related to the severity of illness were found to increase the household financial burden, both for the patient and for caregivers. Health promotion, prevention and early treatment would reduce some of these costs although each country would need to choose the appropriate mix of prevention and treatment according to their relative costs and impact. We also found strong evidence that costs could be reduced by more rational use of medications for NCDs. The costs of medication for all the different types of NCDs considered here accounted for the highest proportion of the direct costs; where addressed, originator brand medicines were frequently used instead of available generics and costs were then substantially higher than they needed to be. While many LMICs already have strategies to promote the rational use of medicines, there is still some way to go particularly in promoting the use of lower cost generics.

The weakness or non-existence of mechanisms to protect households financially from the burden of NCDs is, however, probably the most important finding in this study. In the studies that considered insurance and provided information on reimbursement rates, NCD-related treatment is generally uncommon and frequently patients and their relatives do not report that they claimed any reimbursement from insurance or employers. Likewise, none of the studies we reviewed reported a system of social security that provides compensation for loss of income incurred by patients and their families because of NCDs. Poor households are more likely to suffer disproportionally from the financial effects of this lack of social protection. To meet the costs, households reported taking unsecure loans, using savings or selling household assets, all of which can lead to longer-term problems for the household. For example, the wider literature suggests that many of the loans taken by households for health expenses are at very high interest rates that can take generations to repay [64]. This is part of a bigger problem in LMICs, many of which rely extensively on direct out-of-pocket payments to fund health services. Recently, many have recognized the need to modify the way they raise funds and more generally to modify their health financing systems so as to improve financial risk protection and ensure greater access to needed health services [65]; it is important to note that it will be increasingly necessary to include NCDs in whatever type of financial risk protection strategy is developed. This is particularly important for poor families because NCDs no longer affect only the more affluent people in society [1,8,13,66,67].

While we think that the financial costs reported in this review will overestimate the costs of a typical patient with NCDs, such that the numbers cannot be used to extrapolate the costs of NCDs to a country, they highlight the other consequences of the lack of financial risk protection in LMICs. In the random sample studies, many people with NCDs reported that they did not seek care at all because of financial reasons (Additional file 2). Many of their conditions are likely to become more severe in the absence of treatment, leading to early death and greater problems for caregivers and households. The effects of not seeking care for poorer households is of particular concern given that the ability to work is one of the most important poverty escape routes [68-72]. Strategies to improve financial risk protection will also lead to increased financial access to health services while demand side responses, such as cash transfers, can help reduce some of the financial barriers to seeking care, such as transport costs. Nevertheless, demand-side approaches in LMICs are, to our knowledge, limited largely to maternal and child health (and education) and some communicable diseases [73-77].

Through this review, we are also able to identify areas where further research is needed. Among the four major NCDs, the financial costs from chronic respiratory diseases are very poorly documented, although they cause four times more deaths than for example diabetes, which has been researched more [1,78]. According to the WHO, almost 90% of COPD deaths occur in LMICs and the highest prevalence of smoking – the primary cause of COPD – among men is in these countries [1,78,79]. It would therefore be interesting to have more assessments of the financial costs of these diseases in future studies. Additionally, while all studies reviewed here used cross-sectional data, panel data will be very useful in assessing the evolution of costs incurred by households because of NCDs. The comparison of the relative importance of the cost of NCDs with that of acute illnesses is also of a great interest here, as according to the papers reviewed, there is no clear trend. Indeed, some studies show that NCDs are more costly for households, while others observe the opposite. Sometimes in the same country, different results are found depending on the area (urban vs. rural), the type of health care (outpatient vs. inpatient) and household socioeconomic status (poor vs. better-off) [17,21,38,39,44,55]. More studies – introducing for example a time dimension and a distinction between private and public providers – are therefore needed to shed more light on this issue. It may also be important to expand the geographical outlook in future research to be more representative of a wider group of developing countries. This is true even after accounting for the influence of the languages used in this review. Of the 49 studies found, most were from Asia, as compared to only a handful from Latin America or Eastern Europe, and 10 studies from Africa.

## Conclusions

The literature on the social, financial and economic consequences of NCDs in developing countries has not kept pace with the epidemiological evidence. It has been known for some time that the burden of disease associated with NCDs and injuries is already higher than that associated with the health conditions included in the Millennium Development Goals (HIV/AIDS, tuberculosis, malaria, and maternal, child and reproductive health), even in developing countries. Moreover, it has been well documented that the share of NCDs in the overall disease burden will continue to increase globally. Indeed, the UNs’ 2011 conference on NCDs stressed the importance of these diseases as a development issue.

The literature we reviewed sheds some light on the financial consequences of NCDs on households in LMICs. Nonetheless, there are limitations to generalization of these findings due to methodological challenges. Valid estimates of the average costs of NCDs will require random samples with controls to account for people who have costly and less costly treatments, and what would have happened in the absence of the diseases. Panel data would be ideal although these studies are more expensive than cross-sectional designs. However, importantly, this review suggests that it is equally as important to focus on people who could not seek care for NCDs due to financial reasons. Little is known about the subsequent development of disease, impacts on these people’s health and the financial, social and other consequences associated with foregone treatment.

The push to develop health-financing systems that improve financial risk protection and help achieve universal health coverage in LMICs is promising. However, policymakers need to ensure that the health as well as the financial burden from NCDs is adequately addressed in future reforms, while at the same time improve access and financial protection for all other health services needed by the population.

## Endnotes

^a^US$ 995 or less, US$ 996 to US$ 3,945, and US$ 3,946 to US$ 12,195, respectively.

^b^Defined as out-of-pocket expenses that accounted for ≥30% of the total annual household income that was reported at baseline.

## Abbreviations

COPD: Chronic obstructive pulmonary disease; LMICs: Low- and middle-income countries; NCDs: Non-communicable diseases; NCMS: New cooperative medical scheme; WHO: World Health Organization.

## Competing interests

The authors declare that they have no competing interests.

## Authors’ contributions

PS and KX conceived the project and the questions to be studied. HTK and PS conducted the literature review, produced the draft of the paper and produced the final version with the input of other authors. DBE and KX contributed to writing the paper. All authors read and approved the final manuscript.

## Authors’ information

The views and opinions expressed in this article are entirely those of the authors and should not be attributed in any manner whatsoever to the organizations the authors are affiliated to. HTK is PhD Student at Aix-Marseille University (Aix-Marseille School of Economics), Marseille, France. PS is Technical Officer at the Department of Health Systems Financing, World Health Organization, Geneva, Switzerland. KX is Team Leader, Health Care Financing at WHO Regional Office for the Western Pacific Region in Manila, Philippines. DBE is the Director of the Department of Health Systems Financing, World Health Organization, Geneva, Switzerland.

## Supplementary Material

Additional file 1: Table S1 Studies reviewed. Click here for file

Additional file 2 Financial difficulties: a major cause for not seeking care for NCDs. Click here for file
