# Supplementary material for: The financial burden from non-communicable diseases in low- and middle-income countries: a literature review
Source: Health Res Policy Syst. 2013 Aug 16;11:31. doi: 10.1186/1478-4505-11-31 (PMC3751656; doi:10.1186/1478-4505-11-31)
Supplement: Additional file 1: Table S1 — Studies reviewed. [file 1478-4505-11-31-S1.doc]

**Additional file1: Table S1:** Studies reviewed

| **Reference and country** | **Disease(s)** | **Study population** | **Methods** | **Selected findings** |
| --- | --- | --- | --- | --- |
| Abegunde and Stanciole (2008)  Russia | Chronic diseases | Households covered by the 1997-2004 Life Standards Measurement Survey (LSMS): an average of 4,179 households samples each year | Two-step Heckman estimation and other regression methods (Tobit, OLS-FE, IV-FE, OLS-RE, and IV-RE) | Each additional case of chronic disease in a household increased the probability of incurring health care expenditure by 8%, increased the healthcare expenditure by 6.2% and decreased the household earned (labour) income by 4.8%. |
| Arrossi et al. (2007)  Argentina | Cervical cancer | 120 new cervical cancer patients in Buenos Aires. | Descriptive analysis and a multivariate logistic regression | -Delays in payments for telephone or electricity affected 52 (43%) households and as a result 14 households had the service cut.  -The daily food consumption was reduced in 37% of households and 38% of households had to sale their property or use savings. |
| Atipo-Ibara et al. (2004)  Congo | Cirrhosis | 166 patients hospitalized for the first time for cirrhosis from January 1999 to December 2000 at the University Hospital Centre of Brazzaville | Descriptive analysis | The mean per-patient cost was 272,345 FCFA (415.79 €) in cases involving ascites, 195,675 FCFA (298.74€) in cases involving encephalopathy, 245,680 FCFA (375.08 €) in cases involving gastrointestinal bleeding and 205,615 FCFA (313.90 €) in uncomplicated cases |
| Chatterjee et al. (2011)  Thailand | Diabetes | 475 diabetic patients who received treatment at Waritchaphum hospital in sakhon Nakhon province | Descriptive analysis, unpaired *t*-test, revealed preference method, proxy good method and sensitivity analysis | Caregivers spent on average 42.21 (±39.94) hours per month on health care activities, 9.28±25.90 hours on instrumental activities of daily living, 6.79±15.45 hours on activities of daily living and 5.80±10.13 hours on household activities of daily living |
| [Chow](http://www.ncbi.nlm.nih.gov/pubmed?term="Chow SK"%5BAuthor%5D) et al. (2002)  Malaysia | Rheumatic diseases | 141 patients in 3 rheumatology clinics in the Klang Valley | Descriptive statistics and multiple logistic regression | Among the patients who had used oral traditional medicine, 51% spent less than RM50 per month, 22% spent RM50 – 100, 21% spent RM100 – 500 and 6% spent more than RM500. 51% had a duration of use greater than 2 months. |
| Chuma et al. (2007)  Kenya | Chronic illnesses | 294 rural and 576 urban households, covering respectively 2,162 and 3,125 individuals in Kilifi district.  9 focus group discussions (FGDs) and 9 in-depth interviews with key informants in each setting. | χ^2^ test and Student’s *t*-test | The costs of health care for chronic illness in rural and urban areas represented 5% and 5.7% respectively of household income. The poorest households in both settings incurred the highest cost burdens for all categories of illnesses. |
| de-Graft Aikins (2005)  Ghana | Diabetes | 77 people with diabetes from two urban towns (Accra, Tema) and two rural towns (Nkoranza and Kintampo) | Longitudinal qualitative study with individual interviews, group interviews, and ethnographies | The high cost of biomedical drugs and recommended foods undermined the commitment of low income and financially destitute groups to long term engagement in biomedical care. |
| Dror et al. (2008)  India | Chronic diseases | 3,581 households, representing 17,323 persons, in five locations of Maharashtra, Bihar and Tamil Nadu states in India | Descriptive analysis, non parametric Median test and multivariate linear regression analysis. | -In chronic diseases, the highest median costs were due to hospitalization and drugs (INR 300 each).  -The cost of chronic illnesses and accidents was significantly higher the cost of acute illnesses in direct, indirect and total cost categories. |
| Elrayah et al. (2005)  Sudan | Diabetes (Type 1) | 147 children with type 1 diabetes attending public or private clinics in Khartoum State | Descriptive analysis, χ^2^ test , Student’s *t*-test and Mc Nemar two-sided change test | 65% of the previous year’s family expenditure on health was used for the diabetic child.  The median annual expenditure of diabetes care was US$ 283 per diabetic child, of which 36% was spent on insulin |
| Falconer et al. (2009)  Vanuatu | Type 2 diabetes | 172 patients with known diabetes, drawn from the Port Vila Central Hospital catchment area | Descriptive analysis | The main costs incurred by individuals were accounted for over-the-counter (OTC) medications and transport to and from health care facilities. |
| Goldhaber-Fiebert et al. (2010)  India, China, Thailand and Malaysia | Type 2 Diabetes Mellitus (DM) | -India: 331 patients;  -China: 970 patients in Hangzhou, Zhejiang province and 883 patients from Shandong Province;  -Thailand: 8,596 patients ;  -Malaysia: 100 patients | -Descriptive analyses  -Two-tailed *t*-tests  -ANOVA followed by Dunnett post-hoc test | Average hospital admission expenditure relative to per-capita income among diabetic patients varied from 11% of per-capita national income in Malaysia to 98% in Shandong, and was even more for patients with specific complications. A hospital admission for diabetic patients with complications cost from 12% up to 102% of per-capita income on average. |
| Gombet et al. (2009)  Congo | Cardiovascular diseases | 197 patients admitted for stroke from July to December 2006 in the Emergency Department of the Brazzaville University Hospital | Descriptive analysis and Student’s *t*-test | The drugs used were specialties in all cases and the most expensive test was brain scanner which was charged to 100,000 FCFA (150 €) |
| Gonzalez-Gonzalez et al. (2011)  Mexico | Chronic diseases and disabilities | 48,600 households covering 206,700 individuals, representative of all the population of Mexico | Descriptive analysis, *t* and χ^2^ tests and logistic regression models | Those with chronic diseases were also more likely to face acute morbidity, ambulatory care and hospitalizations, with odds ratios of 2.67 (95%CI: 2.56-2.79), 2.99 (95%CI: 2.86-3.13) and 2.91 (95%CI: 2.74-3.10) respectively |
| Gotsadze et al. (2005)  Georgia | Chronic diseases | 2,500 households covering 9,773 individuals sampled in each of Tbilisi’s 11 districts | t-test, one-way ANOVA, χ^2^ test and multiple logistic regressions | Among poorer households, 30% of those seeking outpatient care stated that they were unable to meet health care costs compared with 11.6% in the richest quintile. |
| Gotsadze et al. (2009)  Georgia | Chronic diseases | 2,859 households, covering 10,445 individuals in Georgia | Descriptive analysis and logistic regression model | The odds ratios of facing catastrophic expenditure were 4.4 and 27 times higher among household having incurred expenses for treating chronically ill persons and those that had case of hospitalization respectively. |
| Goudge et al. (2009)  South Africa | Chronic diseases | 280 households, covering 1,446 individuals in Mpumalanga province | -χ^2^test with data from the household survey;  -A livelihood analysis using data from a qualitative study of 30 selected households over 10 months | For highly vulnerable and vulnerable households, repeat visits generated cost burdens amounting to 30-50% of monthly income, unaffordable without gifts from social networks |
| Grover et al. (2005)  India | Diabetes mellitus | 50 patients aged between 20 to 50 years, with an illness duration of 5–10 years and living with relatives, selected at the diabetic clinic of a large multispecialty teaching hospital in north India | Descriptive analysis, Student’s *t*-test, Spearman’s correlation coefficients tests | The mean total direct treatment cost of diabetes mellitus over the entire six month period amounted to 4,966.42 rupees (S.D.=4,270.42). With 3.076,28 rupees (S.D.=2546.92), representing 61.94% of the mean direct cost, the money spent on drugs was the highest |
| Hao et al. (2010)  China | Chronic diseases | 671 households and 1,877 individuals enrolled in National Pilot Medical Financial Assistance Scheme (MFA) in the counties of Wuxi (relatively poor) and Qianjiang (relatively wealthy) | Two-level linear multilevel model and binomial regression | The presence of chronic disease was one of the factors that had strong association with presence of large amount of medical debt (OR=1.5; 95%CI: 1.1-2.2) |
| Heeley et al. (2009)  China | Cardiovascular diseases (Stroke) | 4,739 patients (aged ≥ 18 years) who had experienced an acute (first-ever or recurrent) stroke as defined by the WHO with no apparent cause other than of vascular origin (hospital registry study) | χ^2^ test, t-test, Mann Whitney U-test, sensitivity analysis, univariate and multivariate logistic regressions. | -A total of 3,384 patients (71%) were estimated to have experienced catastrophic health care costs.  -Catastrophic payments occurred more often in those patients without health insurance (47%) than in those with health insurance (14%) |
| Ir et al. (2010)  Cambodia | “Serious illnesses”, including chronic lifelong conditions | 5,975 households comprising 33,161members in three rural operational health districts (OD) | Descriptive analysis, χ^2^ test, Student’s *t* –test and Mann-Whitney test | Of the 4,992 household members with a serious illness, only 30% said they had received inpatient care. Chronic lifelong conditions led to inpatient treatment in 22% of cases. |
| Kanamura and D’Avila Viana (2007)  Brazil | Chronic diseases | 64,219 beneficiaries of a private health plan | Descriptive analysis, using data from a secondary source | Cancers, circulatory, musculoskeletal and respiratory tract diseases represent 58.1% of diagnoses among high-spender beneficiaries. |
| Kapur, A. (2007)  India | Diabetes | 5,516 persons with diabetes (and on treatment) in the towns and cities constituting a representative sample of Indian population | Descriptive analysis using results of the Cost of Diabetes in India (CODI) study | - Patients without complications had an 18% lower cost while those with three or more complications had a 48% higher cost.  -Overall indirect cost was estimated to be INR 12,756. |
| [Khowaja](http://www.ncbi.nlm.nih.gov/pubmed?term="Khowaja LA"%5BAuthor%5D) et al. (2007)  Pakistan | Diabetes | 345 persons with diabetes selected randomly in six different out-patient clinics at three selected sites in Karachi representing public, private and NGO provision. | Descriptive analysis, Kruskal Wallis and Mann Whitney U tests | - Total mean direct cost borne by the person with diabetes and/or his/her family was estimated to be Rs. 1,930 in the current visit.  - Excluding unemployed persons, the mean lost productivity by study subjects and their attendants was Rs. 113 and 208 respectively and their mean time lost was 3 hours |
| Levinson et al. (2010)  9 low- and middle-income and 10 high-income countries | Mental illness | 44,561 individuals of working age (18-64 years) | Descriptive statistics and two-part regression models using data from World Mental Health (WMH) Surveys conducted by the World Health Organization (WHO) | The model-based simulations estimated that serious mental illness was associated with a reduction in earnings equal to 32% of the median within-country earnings in high-income countries and 33% of median within-country earnings in low- and middle-income countries. |
| Mahmood and Ali Mubashir (2002)  Pakistan | Chronic diseases | 4,021 nationally representative households of which 2,577 were rural and 1,444 urban. | Descriptive analysis | For cancer/ulcers and circulatory diseases, 27.1% and 22.9% respectively used finances from unsecured loans, while 7.1% and 8.8% respectively relied on assistance from others. |
| Mondal et al. (2010)  India | Chronic diseases | 3,150 households in West Bengal, representing 15,277 individuals | Descriptive analysis, simple logit regression and a principle component analysis to develop a Basic Entitlement Index (BEI) | The average household spending on chronic illness in rural areas was Rs. 2,637, which was about 5.73% of household’s total annual expenditure. In urban areas, it was Rs. 3,030, representing 4.14% of household’s total annual expenditure. |
| Mukherjee et al. (2011)  India | Chronic diseases | 543 households covering 2,925 individuals in Kottathara Panchayat | Descriptive analysis and multivariate regression. | The mean annual per capita health expenditure was 233 Rs for a chronic episode and 263 Rs for an acute one. |
| Niëns et al. (2010)  16 Low- and Middle-Income Countries | Asthma, Diabetes, Hypertension and Adult Respiratory Infection | The total population of the 16 countries analyzed amounted to over 775 million people | Descriptive analysis. | Originator brand medicines purchased in the private sector would have impoverished more people compared to the lowest priced generic (LPG) equivalent. |
| Obi and Ozumba (2008)  Nigeria | Cervical cancer | 95 cervical cancer patients seen at the University of Nigeria Teaching Hospital Enugu and who were referred for radiotherapy | Descriptive analysis. | All the patients expressed loss of income from workplaces due to absenteeism, disengagement from work and spending on treatment. |
| Patankar and Trivedi (2010)  India | Bronchitis, asthma, upper respiratory tract infections (URTI), cardiac, other chest illnesses, allergic rhinitis,chronic obstructive pulmonary disease (COPD) | 1,542 individuals above 11 years in six areas of Mumbai | Logistic regression | -Without hospitalization, OOPE of individuals for allergic rhinitis represented 1.7% of the annual personal income when incurred in public healthcare facilities, and 2.7% of annual personal income when incurred in private healthcare facilities. For COPD, these percentages were 10.0% and 13.3% respectively.  -With hospitalization, OOPE for COPD represented 62.3% of the annual personal income when incurred in public healthcare facilities, and 50.7% of annual personal income when incurred in private healthcare facilities. |
| Patel et al. (2007)  India | Depressive disorders, reproductive tract infections (RTIs) and anaemia | 2,494 women aged 18-50 years selected randomly in the catchment area of a primary health centre in the state of Goa | Bootstrapping techniques, percentile method, multivariate model | The mean costs ( lost time costs and out-of-pocket costs) for women with depressive disorders was considerably higher than those for women with anaemia or RTIs, and also greater than those for women without depressive disorders |
| Pepper et al. (2007)  South Africa | Diabetes (hyperglycemic emergency admissions) | 53 diabetic patients admitted to G F Jooste Hospital (GFJH) Cape Town from 1 September to 31 October 2005 | Descriptive analysis | The mean cost per hyperglycaemic admission was R 5,309; this varied according to the type of hyperglycaemic emergency. |
| Ramachandran et al. (2007)  India | Diabetes | 556 type 2 diabetic patients from various urban and rural regions of seven Indian states | Descriptive analysis, χ^2^ test with Yate’s correction, Multiple linear regression | The proportions of income spent on diabetes care by the urban low-, middle-, upper middle-, and high-income groups were 34.0, 16.9, 9.3 and 4.8% respectively. The corresponding percentages in the rural population were 27.0, 12.6, 9.0 and 5.0% respectively. |
| Rao et al. (2011)  India | Cardiovascular diseases (CVD) and diabetes | 2,129 individuals hospitalized for cardiovascular diseases and 438 for diabetes, covering all the 35 States and union territories in India | Descriptive analysis, χ^2^ and *F-* tests | -Overall, the out-of-pocket spending share of annual household consumption expenditure for CVD and diabetes hospitalization was 30% and 17% respectively.  -In the poorest group, OOPS for CVD and diabetes hospitalization consumed 25% of the household consumption expenditure |
| Rayappa et al. (1999)  India | Diabetes | 611 patients selected from government and private institutions in Bangalore | Descriptive analysis | The mean annual direct cost for routine treatment was INR 5,756 for patients of urban areas and 6,266 for those of rural areas. Medicines comprised 32% of this total annual cost for treatment, while consultation represented 38% and transportation 23%. |
| Ross et al. (2007)  Vietnam | Three smooking-related diseases: lung cancer, chronic obstructive pulmonary disease (COPD), and ischaemic heart disease | 390 patients diagnosed with one of the selected illness in 1 district hospital, 1 provincial hospital and 3 national hospitals in Northern Vietnam | Descriptive analysis | Overall, the average cost per admission was $VN5 115,900 ($US341, $US1 = $VN15 000) and was dominated by the out-of-pocket expenditures (83%). Non-medical expenses (travel costs, food, accommodation, informal fees) comprise the largest part of out-of-pocket expenses (61.3%). |
| Ruhweza et al. (2009)  Uganda | Chronic diseases | 384 households in three of the five health sub-districts in Jinja district | Descriptive analysis and logistic regression model | Payment for healthcare was associated with presence of children or someone with chronic illness (OR=1.5; 95%CI:1.3-1.9 and OR=3; 95%CI:1.7-6). |
| Russel and Gilson (2006)  Sri Lanka | Chronic illnesses | 423 households covering 2197 individuals and a longitudinal case study of 16 households in Colombo | Descriptive analysis | Out of the 155 households with a member seeking regular treatment 50% incurred a direct cost burden of 1% of monthly income or less, 87% a burden of 5% or less and only 3% of households incurred regular monthly burdens over 10% |
| Salti et al. (2010)  Lebanon | Chronic diseases | 13,000 households covering 56,000 individuals in all 6 Lebanese governorates | Descriptive analysis, concentration indexes and regression analysis | The presence of a disability and the presence of a chronic condition were associated with significantly higher odds of catastrophic payments |
| Sari et al. (2001)  Kazakhstan | Chronic diseases | 7,073 individuals constituting a nationally representative sample | A two-part regression model | On average, people with cardiac problem, chronic illness or intestinal disorders paid 24%, 18% and 38% more, respectively, than others with other health problems. |
| Shi et al. (2010)  China | Chronic diseases | 3,340 households covering 11,252 individuals in Hebei and Shaanxi provinces, and the Inner Mongolia Autonomous Region | Descriptive analysis and logistic regression | -Household chronic disease proportion was a statistically significant determinant of impoverishment before reimbursement  -Households with chronic disease were less able to escape from impoverishment after reimbursement. |
| Shobhana et al. (1999)  India | Diabetes | 596 patients who received care for diabetes in a Government General Hospital (GGH) and a Private Hospital for Diabetes mellitus (PHD) in the metropolitan city of Madras | χ^2^ test with Yates’ correction, Student’s *t*-test, Median test and Wilcoxon Matched-pairs signed-ranks test. | -Those who visited the PHD from middle and low-income groups spent a considerable proportion of their income on diabetes care: 15.4% and 24.5% respectively;  -Subjects with shorter duration of diabetes spent significantly less than those with longer duration for medical consultation and drugs. |
| Su et al. (2006)  Burkina-Faso | Chronic diseases | 774 households of Nouna ( urban and rural) | Descriptive analysis and multivariate logistic regression | The presence of a member with chronic illness in a household increased the probability of catastrophic consequence by 3.3 to 7.8 times, depending on the threshold. |
| Sun et al. (2009)  China | Chronic diseases | 6,147 rural households, including 3,944 chronic disease patients in Ningxia Autonomous Region and Shandong Province | Descriptive analysis, χ^2^ and t tests | About 15% of families with NCMS membership in Shandong and 14% in Ningxia spent over 40% of their annual non-food expenditure on treating chronic diseases in 2005-2006; these proportions were lower than those of non-NCMS members (21% and 18% respectively) |
| [Thomas](http://www.ncbi.nlm.nih.gov/pubmed?term="Thomas SV"%5BAuthor%5D) et al. (2001)  India | Epilepsy | 285 patients from epilepsy centers attached to University hospitals in Kerala, Tamil Nadu, Karnataka, Andhra Pradesh, Maharashtra, and Gujarat states | Descriptive analysis | The total annual cost per patient amounted to INR 13,755 (USD 344). The direct cost was INR 3,725 (USD 93), and the indirect cost was INR 10,031 (USD 251). |
| Thuan et al. (2006)  Vietnam | NCDs (diabetes, goiter, cancer, neurological problems, rheumatologic problems, hypertension, heart illness, allergic problems) | 621 households covering a total of 2,727 individuals, in the Bavi district of Ha Tay province | Descriptive analysis | -Non-communicable diseases accounted for 27.7% of the total curative expenditures, and communicable illnesses for 60.6%.  -Only 7 households had catastrophic health care expenditure and for these households, non-communicable diseases counted for 11.1% of the total health care expenditure.  -Non-communicable illnesses represented 58.6% of health care expenditure in the group of 12 households having health expenditure between 30% and 40% of households’ capacity to pay. |
| [Villarreal-Ríos](http://www.ncbi.nlm.nih.gov/pubmed?term="Villarreal-Ríos E"%5BAuthor%5D) et al. (2000)  Mexico | Diabetes Mellitus | 258 patients of the Mexican Institute of Social Security (IMSS) from the metropolitan area of Monterrey | Descriptive analysis | Average annual cost per diabetic patient was $708 USD, and the most expensive service was family medicine, at $265 USD |
| [Yip](http://www.ncbi.nlm.nih.gov/pubmed?term="Yip W"%5BAuthor%5D) and [Hsiao](http://www.ncbi.nlm.nih.gov/pubmed?term="Hsiao WC"%5BAuthor%5D) (2009)  China | Chronic diseases | 5,380 households enrolled in the Medical Savings Account (MSA) and the Rural Mutual Health Care (RMHC) in western and Central China | A static simulation model | 11.6% of the impoverished under the US$1.08 poverty line, and 11% of those under the 663 RMB poverty line, became impoverished due to outpatient expenses associated with chronic diseases;  Medical expenditure increased the poverty headcount by 40.7% for chronic disease patients, compared to 22.86% for patients without chronic conditions. |
| [Zhou](http://www.ncbi.nlm.nih.gov/pubmed?term="Zhou B"%5BAuthor%5D) et al. (2008)  China | Cancer | 1,631 cancer inpatients (from medical records) at the First Affiliated Hospital of Nanjing Medical University | Descriptive statistics, Pearson chi-square test, Fisher’s exact test, Mann-Whitney U-test and a multiple linear regression | The median drug cost was approximately 8,069 yuan per inpatient. Among the seven cancers, the median drug cost was highest for esophageal cancer (11,028 yuan) and lowest for breast cancer (4,309 yuan). The drug cost for SHI inpatients (8,933 yuan) was significantly higher (p < 0.001) than that for non-SHI inpatients (7,616 yuan) |
