# Supplementary material for: The financial burden from non-communicable diseases in low- and middle-income countries: a literature review
Source: Health Res Policy Syst. 2013 Aug 16;11:31. doi: 10.1186/1478-4505-11-31 (PMC3751656; doi:10.1186/1478-4505-11-31)
Supplement: Additional file 2 — Financial difficulties: a major cause for not seeking care for NCDs. [file 1478-4505-11-31-S2.doc]

**Additional file 2**

The extent of the household financial burden from NCDs is probably underestimated from the literature. In fact, the problem of access to health care for NCDs should be taken into account to better understand the whole impact of these illnesses on households’ finances. In some studies, significant proportions of patients are found to not seek care at health facilities because of financial reasons (see Appendix Table 2). We can anticipate that economic impact of not accessing care will inherently be considerable for these types of illness that tend to be chronic. The effects of not seeking care for poorer households may be even more worrying because of linkages between health and wealth. Indeed lower income generation capabilities linked to untreated NCD will lead further into the cycle of poverty. This is something we need to study further to have a more precise understanding of the mechanisms of impact as well as coping, but at the same time, policy needs to try to eliminate the lack of access to health services for NCDs due to financial barriers.

**Additional file 2: Table S2:**  NCDs care-seeking behavior in some studies

| **Reference** | **Country** | **Core findings on financial barriers for seeking health care for NCDs** |
| --- | --- | --- |
| Shi *et al.*, 2010 | China | 34.3% of the individuals referred for hospital admission did not seek inpatient services after the doctor’s referral. Among them, 80.2% reported economic barriers as the reason for not seeking inpatient care.  The proportion of respondents who had their need for in-patient admission unmet was significantly associated with proportion of persons in household with chronic illness (4.56 – 10.27 times higher) |
| Hao *et al.*, 2010 | China | 29.5% of the respondents with illness (chronic and acute) in last 2 weeks did not visit physicians for outpatient services, and 61.6% of those who ought-to-use hospitalization services did not use them.  The leading reason for no-use of health services was financial difficulties (80% for out-patient services and 92% for in-patient services. |
| Goudge *et al.*, 2009 | South Africa | No action was taken for 38% of individuals who reported one or more health problems and higher levels of non-consultation were associated with chronic (48%) rather than acute illnesses (9%).  Household inability to pay for transport and hospital fees was one of the most common reasons that explained the failures in the referral system across the three livelihood groups. |
| Gotsadze *et al.*, 2009 | Georgia | In 2007, 42.3% of those suffering from chronic illnesses did not seek care (47.7% in the poorest quintile), compared to 46.7% in 2000 (57.1% in the poorest quintile).  The main reason for not seeking care was financial considerations. |
| Chuma *et al.*, 2007 | Kenya | Rural households were more likely not to seek treatment for chronic conditions than urban households (56.9% and 44.9% respectively; P<0.001).  Lack of cash was the main factor that prevented people from seeking treatment for both acute and chronic illnesses in both settings |
| Gotsadze *et al.*, 2005 | Georgia | 11% of the reported cases of illness (both acute and chronic) were not treated. Among them, 39% were due to the cost of treatment (too expensive). |
| Sari *et al.*, 2001 | Kazakhstan | Of the 677 respondents who answered that they held prescriptions for medicine, almost 42% of them reported that they failed to buy the prescribed drugs when they were sick.  30% of the poor reported they could not afford the prescribed drug. 25% of non-poor reported the same difficulty. |
